# Diagnostic Performance of Interferon-Gamma Releasing Assay in HIV-Infected Patients in China

**DOI:** 10.1371/journal.pone.0070957

**Published:** 2013-08-02

**Authors:** Yanhua Yu, Xiuying Zhao, Wen Wang, Hao Wu, Ming Chen, Wenhao Hua, Huizhu Wang, Ting Wei, Yanmei Jiao, Guizhen Sun, Wei Li

**Affiliations:** 1 Department of Clinical Laboratory, Beijing You’an Hospital, Capital Medical University, Beijing, China; 2 Center for Infectious Diseases, Beijing You’an Hospital, Capital Medical University, Beijing, China; 3 Department of Clinical Laboratory, Beijing Ditan Hospital, Capital Medical University, Beijing, China; 4 Molecular Virology Group, Queensland Institute of Medical Research, Brisbane, Australia; Chinese Academy of Medical Sciences, China

## Abstract

**Background:**

Active tuberculosis infection represents a very common and significant threat to HIV-infected patients. But measures to accurately detect it are limited.

**Objective:**

To compare and analyze the diagnostic efficacy of T-SPOT.TB alone and in combination with TST in HIV-infected patients in China.

**Method:**

TST (tuberculin skin test) and T-SPOT.TB were performed on 131 HIV-infected patients admitted in Beijing You’an Hospital and Beijing Ditan Hospital between Oct, 2010 and Jul, 2012, who were initially diagnosed as suspected ATB (active TB). The patients were further categorized into ATB and Not ATB based on clinical and cultural evidences. The performance of TST and T-SPOT.TB were analyzed and compared.

**Results:**

The sensitivity and specificity of T-SPOT.TB were 41.3% and 94.6%, respectively, both higher than TST (12.9% and 91.8%). By combining T-SPOT.TB and TST, the sensitivity did not increase, but specificity was elevated to 100%. TST, T-SPOT.TB and their combinations all performed better in patients with extra-pulmonary diseases than with pulmonary disorders. False-positive T-SPOT.TB results were found to be associated with history of prior TB. In addition, concomitant bacterial infections and low CD4 counts were associated with increased ATB risk.

**Conclusions:**

T-SPOT.TB is superior in screening ATB in HIV-infected patients in China over traditional TST. Additional TST would help to confirm a positive T-SPOT.TB result. Both tests work better for patients with extra-pulmonary conditions.

## Introduction

Tuberculosis (TB) is one of the most historical and common infectious diseases in the world. Despite the widely used Bacille Calmette-Guerin (BCG) vaccine, around one third of the global population are infected with *Mycobacterium tuberculosis* (*M.TB*) according to the report from the World Health Organization (WHO) [1]. Most of the TB patients are in developing countries, including China. The incidence of active tuberculosis (ATB) is doubled and death rate rises 7-fold in the past decade in China [2].

The risk of the progression of latent TB infection to active diseases is around 5–10% [3]. And the most important risk factor is HIV infection [4], [5]. The risk of progression to active TB is 10% in HIV-infected patients [6].

Until the development of interferon-gamma releasing assays (IGRAs), tuberculin skin test (TST) was used to screen TB. TST has its advantages to relatively accurately predict the risk of developing active TB from latent TB infection [7], [8]. But, false positive results could be seen, especially in BCG-vaccinated population, and patients infected with *Nontuberculous Mycobacteria* (NTM), while false negative results are very common in immuno-compromised population. And it takes two visits to complete the test [9]. The new generation IGRAs offer superior sensitivity and specificity over TST by using multiple specific antigens (e.g. ESAT-6 and CFP10) exclusively in *M.TB* but not BCG and NTM [10], [11], [12], [13], [14], [15], [16]. Currently, there are two IGRAs available commercially (QuantiFERON-TB Gold in Tube test [QFT] and T-SPOT.TB [TSPOT] [17], [18].

In HIV-infected patients, the rate of positive TST and TB DNA PCR is very low, much lower than in immunocompetent population, possibly due to compromised immune system [9]. Clinical manifestations of ATB in HIV-positive patients vary significantly from those for the immunocompetent population. Diagnosing active TB in HIV-infected patients, despite of incorporation of clinical, radiological and pathological measures, is difficult, and only relies on microbial culture and acid-fast staining, whose sensitivity is not high in ATB [19]. A rapid and reliable diagnostic tool for TB in AIDS patients is much needed.

The use of IGRAs as a diagnostic tool for latent TB infection has been approved by the US Center for Disease Control and Prevention (CDC) [18]. But the data on the use of IGRAs on HIV-infected patients is very limited [20], [21].

Here we analyzed the efficacy and accuracy of using T-SPOT.TB to diagnose ATB in HIV-infected patients, and compared the results with TST.

## Materials and Methods

### Study Population

This study has been approved by Beijing You’an/Ditan Joint Institutional Review Board, Beijing You’an/Ditan Joint Ethics Committee, Capital Medical University. Written informed consent was obtained from each participant enrolled in this study. A prospective study was conducted in HIV-positive patients admitted in the Center for Infectious Diseases, Beijing You’an Hospital, and Department of Infectious Diseases, Ditan Hospital, Capital Medical University, between Oct, 2010 and Jul, 2012. Patients who had received anti-TB therapy within 1 year before enrollment were excluded. A total of 131 patients who had a suspected or confirmed ATB diagnosis were enrolled and tested with TST and T-SPOT.TB. There were 7 patients who died within 3 months after enrollment, 7 who lost follow up, 4 without a confirmed final diagnosis, and 3 with a incomplete T-SPOT.TB. These 21 subjects were excluded from analysis. There were 120 subjects who were included in the analysis. TST and T-SPOT.TB were all performed simultaneously upon enrollment. All patients were followed up for 3 months.

### Definitions and Diagnosis

TB suspects were defined as patients with clinical or radiographic manifestations consistent with the diagnosis of ATB, but absence of microbial culture or pathological evidences [22]. The patients were categorized into 2 groups based on diagnosis: (1) ATB: final diagnoses were based on clinical manifestations and radiographic evidence with or without positive culture or pathological results; (2) Not ATB: failed to meet above two categories and clinical manifestations subsided without TB treatment.

### TST

TST (tuberculin skin test) was performed by a trained and skilled nurse blinded to patients’ information. Five tuberculin unit (TU) of PPD-S (Statens Seruminstitut, Copenhagen, Denmark) was intradermally injected on the patients’ forearm. The size of the induration was read 72 hours after injection. The cut-off value of induration diameter for positive (+) is > = 5 mm.

### T-SPOT.TB

T-SPOT.TB kit was purchased from Oxford Immunote Ltd. (Oxford, UK) and performed according to manufacturer’s instructions as previous described [23] by a trained technician blinded to patients’ medical information, including TST and culture results. Briefly, peripheral blood mononuclear cells (PBMCs) were collected and plated with a minimum of 2.5×10^5^ cells per well in plates coated with anti-interferon-γ antibodies and cultured at 37°C for 16–20 hours, after which an enzyme-conjugated secondary antibody and chromogenic substrate were applied to generate color spots. The spots were then read and scored using an ELISPOT plate reader (AID-Gmb-H, Germany).

### Statistical Analysis

SPSS software (version 15.0) was used for data analysis. Student's *t* test and the Wilcoxon rank-sum test were used for analysis of continuous variables. Continuous variables were assessed for normality and presented as a mean ± SEM. Categorical variables were compared with the chi-square test or Fisher’s exact test. A p values less than 0.05 was considered statistically significant. Ninety five percent confidence intervals (95%CI) were estimated according to the binomial distribution. Concordance between the results of TST and T-SPOT.TB was assessed using κ coefficients (κ, 0.7, excellent agreement; 0.4, poor agreement; 0.7–0.4, fair to good agreement).

## Results

### Patient Characteristics

Of 120 enrolled HIV(+) patients who were ATB suspects with a valid T-SPOT.TB result, 76 (63.3%) had pulmonary diseases and 44 (36.7%) had not. All patients were vaccinated with BCG. Patients who had both pulmonary and extra-pulmonary diseases were grouped into the pulmonary disease group. There were 46 (38.3%) patients who were diagnosed as ATB. The others were defined as Not ATB (74/120, 61.7%). Clinical characteristics of enrolled patients were shown in **Table 1**. The comorbidities were defined as infections caused by pathogens other than TB and HIV. Viral infections included HBV, HCV, and CMV infections diagnosed by detection of specific antibodies or viral genes. The bacterial infections were defined by isolation of pathogenic bacteria strains or responsiveness to antibiotic therapy. PCP (Pneumocystic pneumonia) was diagnosed by isolation of PCP strains or responsiveness to PCP specific therapy combined with positive chest radiographic findings.

**Table 1:** Clinical characteristics of 120 patients with suspected ATB.

	Total (n = 120)	ATB (n = 46)	Not ATB (n = 74)
Age	40.5±12.8	40.2±7.5	40.7±15.4
Male/Female	110/10	39/7	71/3
Presence of TB history	15	8	7
Presence of TB contact	33	19	14
Comorbidities			
Viral infections (other than HIV)	18	10	8
Bacterial infections	31	25*	6
PCP	34	24	10
Received HAART	51	23	28

ATB, active tuberculosis; Not ATB, diagnosis other than active tuberculosis; CXR, Chest X-ray; HAART, Highly active antiretroviral therapy; PCP, Pneumocystic pneumonia; *p<0.05 if compared with Not ATB group.

The average age of all suspected ATB patients were 40.5±12.8 years old, and most of them were male (110/120, 91.7%). Among all comorbidities, the bacterial infections were positively associated with ATB in HIV(+) patients (p = 0.026). PCP and bacterial infections were not related to ATB statistically (both p>0.05).

The laboratory findings of the suspected ATB patients were demonstrated in **Table 2**. The CD4 cell counts were linked to ATB negatively if compared with the Not ATB group (p = 0.025).

**Table 2:** Laboratory findings of 120 patients with suspected ATB.

	Total (n = 120)	ATB (n = 46)	Not ATB (n = 74)
ESR (mm/h)	70.13±37.9	73.5±40.2	67.8±36.6
RBC (×10^12^/L)	3.61±0.9	3.28±1.0	3.8±0.8
WBC (×10^9^/L)	5.29±2.7	5.19±2.5	5.4±2.8
Neutrophils(×10^9^/L)	37.6±34.9	37.2±36.5	37.8±34.3
Eosinophils(×10^9^/L)	2.11±5.0	1.4±3.6	2.6±6.8
Lymphocytes(×10^9^/L)	12.55±14.3	12.9±13.4	12.3±15.0
CD4 (/mm^3^)	117.35±140.0	92.5±130.9*	134.7±146.3

ATB, active tuberculosis; Not ATB, diagnosis other than active tuberculosis; ESR, erythrocyte sedimentation rate; WBC, white blood cells; RBC, red blood cells; *, p<0.05 if compared with Not ATB group.

### Diagnostic Performance of T-SPOT.TB by Different Infection Sites

The performance of TSPOT for the 120 patients is presented in **Table 3**. The sensitivity and specificity of TSPOT were 41.3% (95%CI, 35.4%–48.8%) and 94.6% (95%CI, 84.9%–99.3%), respectively. PPV, NPV, LR+ and LR- were also calculated and presented in **Table 3**.

**Table 3:** Diagnostic performance of TSPOT.TB.

Parameter	Value	95%CI
Sensitivity, %(n)	41.3%	35.4%–48.8%
Specificity, %(n)	94.6%	84.9%–99.3%
PPV, %(n)	81.3%	74.2%–87.3%
NPV, %(n)	71.8%	65.1%–78.9%
LR+	2.89	1.57–3.49
LR−	0.26	0.18–0.35

PPV, positive predictive value; NPV, negative predictive value; LR+, likelihood ratio for positive test; LR−, likelihood ratio for negative test.

We further analyzed the results by infection sites (**Table 4**). The sensitivity of T-SPOT.TB for pulmonary TB was significantly lower than that for extra-pulmonary TB (38.2% (95%CI, 30.8%–47.3%) vs. 66.7% (95%CI, 61.9%–74.5%), p = 0.023), and the specificity for both sites was similar (92.9% (95%CI, 83.2%–97.7%) vs. 96.9% (95%CI, 91.3%–99.2%)). Next, extra-pulmonary TB infections were further stratified. The best performance was seen in CNS TB infections, where both sensitivity and specificity were 100% and 90.0%, respectively.

**Table 4:** Comparison of performance of T-SPOT.TB in pulmonary and extra-pulmonary TB.

	ATB (n)	Not ATB	T-SPOT.TB (+)	T-SPOT.TB (−)	Sensitivity (95%CI)	Specificity(95%CI)
Pulmonary	34	42	16	60	38.2% (30.8%–47.3%)	92.9% (83.2%–97.7%)
Extrapul	12	32	8	36	66.7% (61.9%–74.5%)	96.9% (91.3%–99.2%)
CNS	4	10	4	10	100%	90.0%
Pleurisy	1	0	0	1	N/A	N/A
Abdominal	1	3	0	4	0%	66.7%
Lymphadenitis	4	4	3	5	50%	50%
Other sites	2	15	1	16	50%	93.3%
Total	46	74	24	96	41.3% (35.4%–48.8%)	94.6% (84.9%–99.3%)

Extrapul, extra-pulmonary conditions; CNS, central nerve system; ATB, active tuberculosis; Not ATB, diagnosis other than active tuberculosis; N/A: non-calculable.

### Risk Factors for False-positive T-SPOT.TB Results

Next we tried to investigate the possible risk factors for false T-SPOT.TB results. A number of potential risk factors associated with false-positive and false-negative T-SPOT.TB results were analyzed and calculated by multivariate analysis. Only the history of prior TB was found to be associated with false-positive T-SPOT.TB results (OR = 3.89, 95%CI = 1.11–8.28, and p = 0.034). No risk factors were found for false-negative T-SPOT.TB results (all p>0.05).

### Comparison between T-SPOT.TB and TST

TST was used on all patients and the performance was demonstrated in **Table 5**. Among all 120 tested patients, there were only 12 (10.0%) tested positive. The overall sensitivity and specificity were 12.9% and 91.8%, respectively. The PPV and NPV were 50.0% and 62.5%, respectively. The sensitivity of T-SPOT.TB was significantly higher than that of TST (41.3% vs. 12.9%). The specificity of the two tests was comparable. The concordance between TST and T-SPOT.TB was measured by kappa coefficient (κ), and poor agreement was found (κ = 0.135).

**Table 5:** Diagnostic performance of the combination of T-SPOT.TB and TST.

	Sensitivity	Specificity	PPV	NPV
TST	12.9%	91.8%	50.0%	62.5%
T-SPOT.TB	41.3%	94.6%	81.3%	71.8%
TST+ T-SPOT.TB(parallel)^a^	45.2%	85.7%	66.7%	71.2%
TST+ T-SPOT.TB(serial)^b^	9.7%	100%	100%	63.6%

PPV, positive predictive value; NPV, negative predictive value; TST, tuberculin skin test; ^a^, parallel: either TST or T-SPOT.TB is positive; ^b^, serial: both TST and T-SPOT.TB are positive;

### Diagnostic Efficiency of the Combination of T-SPOT.TB and TST

Since neither T-SPOT.TB nor TST demonstrated good sensitivity and specificity, we further evaluated the diagnostic efficiency of the combination of T-SPOT.TB and TST by parallel and serial testing algorithms. The results were shown in **Table 5**. Comparing with single T-SPOT.TB, the parallel testing of T-SPOT.TB and TST gave a little better sensitivity (45.2% vs. 41.3%), but a much worse specificity and PPV (85.7% vs. 94.6%, and 66.7% vs. 81.3%, respectively). The sensitivity of serial testing was the lowest (9.7%) among all single and combination testing. But serial testing offered perfect specificity and PPV (both 100%).

We further categorized the enrolled subjects based on their infection sites into pulmonary and extra-pulmonary groups, and compared their efficacy of single and combination of T-SPOT.TB and TST (**Table 6**). In both groups, the patterns of the sensitivity, specificity, PPV and NPV among different testing algorithms were similar to those in pooled subjects. The only exception was found in extra-pulmonary group, where parallel testing had a worse sensitivity than T-SPOT.TB alone (62.5% vs. 66.7%). In addition, we found that all testing algorithms performed worse in pulmonary group and better in extra-pulmonary group if compared with their performance in pooled subjects. (**Table 6**).

**Table 6:** Diagnostic performance of the combination of T-SPOT.TB and TST by infection sites.

		Sensitivity	Specificity	PPV	NPV
Pulmonary (n = 76)	TST	8.7%	88.9%	40.0%	53.3%
	T-SPOT.TB	38.2%	92.9%	81.8%	64.1%
	TST+ T-SPOT.TB (parallel)^a^	39.1%	81.5%	64.3%	61.1%
	TST+ T-SPOT.TB (serial)^b^	8.7%	100%	100%	56.3%
Extrapul (n = 44)	TST	25.0%	95.5%	66.7%	77.8%
	T-SPOT.TB	66.7%	96.9%	80.0%	84.0%
	TST+ T-SPOT.TB (parallel)^a^	62.5%	90.9%	71.4%	87.0%
	TST+ T-SPOT.TB (serial)^b^	12.5%	100%	100%	75.9%

Extrapul, extra-pulmonary conditions; PPV, positive predictive value; NPV, negative predictive value; TST, tuberculin skin test; ^a^, parallel: either TST or T-SPOT.TB is positive; ^b^, serial: both TST and T-SPOT.TB are positive.

## Discussion

Detecting tuberculosis infections in HIV-infected patients is difficult, since the numbers of CD4+ and CD8+ cells were depleted in these patients, and these types of cells were critical in controlling TB infections [20]. TST is a T-cell-based test. Depletion of T cells would affect its performance greatly [20]. Other traditional approaches such as TB DNA PCR are also not sensitive and reliable [10]. The blood and sputum cultures are time-consuming and come with significant false-negative results [9], [10]. T-SPOT.TB is designed to detect the secretion of interferon-gamma by T cells. In HIV-infected patients, its performance may also be reduced by T-cell depletion. Cattamanchi et al did a detailed meta-analysis at 2011 and demonstrated the affected performance of TSPOT.TB in different HIV-infected population [24]. Among all tests available, T-SPOT.TB has demonstrated its great potential on diagnosing both pulmonary and extra-pulmonary tuberculosis [19]. Studies have been done to determine its accuracy in latent and active TB, and the results showed that TSOPT.TB failed to distinguish latent and active TB [15]. However, its detailed role in determining ATB in HIV-infected patients are inconclusive [24].

Our data showed that T-SPOT.TB had a poor overall sensitivity and an acceptable specificity in HIV-infected patients (41.3% (95%CI, 35.4%–48.8%)) and 94.6% (95%CI, 84.9%–99.3%), respectively) (**Table 3**). The sensitivity is significantly poorer but the specificity is higher than those reported in immunocompetent population (94.7% and 84.1%, respectively) [19]. This is also lower than the sensitivity from a meta-analysis report, in which the pooled sensitivity for HIV-infected patients was 72% in low/middle-income countries [24].

We further stratified the results based on the infection sites (**Table 4**). It appears that the sensitivity of T-SPOT.TB for pulmonary TB was much lower than for extrapulmonary TB (38.2% vs. 66.7%). These data are different from what was observed in immunocompetent population, in which sensitivities for both types of TB were over 90% (95.6% and 93.3%, respectively) [19]. The specificity of T-SPOT.TB in our study was 92.9% for pulmonary TB, much higher than in immunocompetent population (69.2%). The specificity for extra-pulmonary TB was also higher than in immunocompetent population (96.9% vs. 88.9%) [19]. The causes of these differences are unclear. It is possible that, in immune deficient patients, the release of IFNγ is limited, and therefore the positive rate of T-SPOT.TB is reduced. For CNS TB, the sensitivity and specificity of T-SPOT.TB were 100% and 90.0% (**Table 4**). This is consistent with previous data published on immunocompetent population, where were 100% and 88.5% [19]. We also found that TST, T-SPOT.TB and their combinations all performed better in extra-pulmonary group than in pulmonary group (**Table 6**). It may be because that the patients with extra-pulmonary TB had a more advanced disease and therefore easier to be detected. Further studies are required to clarify it.

The agreement between TST and T-SPOT.TB results in HIV-infected patients has been investigated based on kappa (κ) value in two previous studies [24]. In low/middle-income countries, the results were poor or moderate (κ = 0.4–0.6) [24]. Our results showed that the kappa value was even lower (κ = 0.135) indicating worse agreement between TST and T-SPOT.TB compared with previous studies.

Since both TST and T-SPOT.TB failed to give accurate and reliable results alone in detecting TB infections among HIV-infected patients, we further evaluated the efficacy of their combination (**Table 5**). Based on our results, combination did not improve sensitivity if compared with TSPOT alone. T-SPOT.TB alone had the best sensitivity (81.3%) and NPV (93.9%). Parallel testing offered best sensitivity (45.2%), but lowest specificity (85.4%) among all single and combinational testing strategies. TST alone had a very low sensitivity (12.9%), but a high specificity (91.8%). T-SPOT.TB performed better than TST in term of every parameter. Taking together, we believe that, in HIV positive patients, T-SPOT.TB alone would be a good choice to screen for ATB. To confirm a positive T-SPOT.TB result, additional TST should be performed.

There are potential limitations in this study. For example, the size of the study population is not very large and the results of statistical analysis are, therefore, may need further confirmation. In addition, most of the subjects in this study were male (110/120, 91.7%). The gender may post as a confounding factor to the statistical results.

In conclusions, we demonstrated the superior capability of T-SPOT.TB to screen for ATB in HIV-infected patients in China over traditional TST. Additional TST would increase specificity, but do not improve sensitivity.
